# Diabetes and risk of cancer incidence: results from a population-based cohort study in northern Italy

**DOI:** 10.1186/s12885-017-3696-4

**Published:** 2017-10-25

**Authors:** Paola Ballotari, Massimo Vicentini, Valeria Manicardi, Marco Gallo, Sofia Chiatamone Ranieri, Marina Greci, Paolo Giorgi Rossi

**Affiliations:** 1 Epidemiology Unit, Local Health Authority of Reggio Emilia, IRCCS, Reggio Emilia, Italy; 2 Department of Internal Medicine, Hospital of Montecchio, Local Health Authority of Reggio Emilia, Reggio Emilia, Italy; 3 Oncological Endocrinology Unit, AOU Città della Salute e della Scienza di Torino, Turin, Italy; 4 Clinical Pathology and Microbiology Laboratory, Department of Laboratory Medicine, G. Mazzini Hospital, Local Health Authority of Teramo, Teramo, Italy; 5 Primary Health Care, Local Health Authority of Reggio Emilia, Reggio Emilia, Italy

**Keywords:** Diabetes mellitus, Cancer incidence, Diabetes registry, Cancer registry, Diabetes treatment, Oral hypoglycaemic agents, Insulin

## Abstract

**Background:**

Aim of this study was to compare cancer incidence in populations with and without diabetes by cancer site. Furthermore, we aimed at comparing excess risk of cancer according to diabetes type, diabetes duration and treatment, the latter as regards Type 2 diabetes.

**Methods:**

By use of the Reggio Emilia diabetes registry we classified the resident population aged 20–84 at December 31^st^ 2009 into two groups: with and without diabetes. By linking with the cancer registry we calculated the 2010–2013 cancer incidence in both groups. The incidence rate ratios (IRR) by cancer site, type of diabetes, diabetes duration, and as concerns Type 2 diabetes, by treatment regimen were computed using Poisson regression model and non-diabetic group as reference.

**Results:**

The cohort included 383,799 subjects without diabetes and 23,358 with diabetes. During follow-up, we identified 1464 cancer cases in subjects with diabetes and 9858 in the remaining population. Overall cancer incidence was higher in subjects with diabetes than in those without diabetes (IRR = 1.22, 95%CI 1.15–1.29), with similar results focusing on subjects with at least 2-year diabetes duration. Cancer sites driving overall increased risk were liver, pancreas, Colon rectum, and bladder in both sexes, corpus uteri for females. There was also suggestion of an increased risk for kidney cancer in females and a decreased risk for prostate cancer. Excess risk was found in patients with Type 2 diabetes, more marked among insulin users, especially with combined therapy.

We observed an increasing risk for diabetes duration up to 10 years from diagnosis (IRR = 1.44, 95%CI 1.29–1.61) and a subsequent decrease to moderate-higher risk (IRR = 1.15, 95%CI 1.04–1.30).

**Conclusions:**

Our study indicates that the strength of association depends on specific cancer site. Insulin, monotherapy or combined therapy, per se or as an indication of poor blood glucose control, in addition to diabetes duration, may play a role in the association of diabetes and cancer.

**Electronic supplementary material:**

The online version of this article (doi:10.1186/s12885-017-3696-4) contains supplementary material, which is available to authorized users.

## Background

Diabetes is a major public health concern worldwide. It is increasing at an alarming rate. In 2013, 382 million people had diabetes and there will be 592 million by 2035 [1].

There is considerable evidence linking diabetes and cancer incidence [2, 3] and many epidemiological studies have found association between diabetes and several types of cancer, such as liver, pancreas, endometrium, kidney, breast, prostate, bladder, and colorectal cancer [4–11]. Hyperglycaemia and hyperinsulinaemia are most reliable hypotheses of potential biological mechanisms linking diabetes and cancer [12], with the latter being more consistent [13]. For some type of cancers, insulin and oral hypoglycaemic agents (OHAs) may also represent risk or protective factors, although evidence is inconclusive [12, 14, 15]. The Diabetes and Cancer Research Consortium recommended high-quality observational studies to avoid time bias and to take into account various confounding and modifying factors, in order to better understand the association between diabetes mellitus and cancer incidence, in addition to the potential role of glucose-lowering treatment [12].

Many studies and systematic reviews have compared cancer incidence between diabetic patients and general population, but only few analyzed an entire population of subjects with known diabetes. Considering that Reggio Emilia province relies on both cancer and diabetes registry, we could design and conduct a population-based cohort study.

Aim of this study was to compare cancer incidence in populations with and without diabetes by cancer site. Furthermore, we aimed at comparing excess risk of cancer according to diabetes type, diabetes duration and treatment, the latter as regards Type 2 diabetes.

## Methods

### Setting and study population

**Fig. 1:**
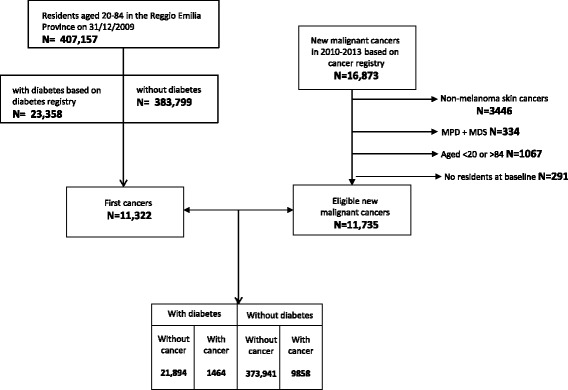
Flowchart portraying the linkage process for all sites analysed. MPD = chronic myeloproliferative disorders; MDS = myelodysplastic syndromes

The study cohort included the inhabitants of the Reggio Emilia province (Northern Italy) who were aged 20–84 on December 31^st^, 2009. According to Reggio Emilia diabetes registry (accessed in July 2013), we classified the entire resident population as with or without diabetes (Fig. 1).

The methods applied to set up the diabetes registry have been previously described [16]. In brief, diabetes registry was created by deterministic linkage of six routinely collected data sources through a definite algorithm able to ascertain cases and to distinguish types of diabetes (Type 1, Type 2 and secondary diabetes, i.e. diseases of the exocrine pancreas and drug-induced diabetes), treatment, and care model (for Type 2 diabetes). We used routinely collected health databases covering hospital discharge, drug dispensation, biochemistry laboratory (for glycated haemoglobin), disease-specific exemption, diabetes outpatient clinics activity, and mortality. Women affected by gestational diabetes or those receiving metformin for polycystic ovary syndrome were excluded from the registry. Cases initially notified to the registry through record linkage are retained in case they were clinically confirmed by a diabetologist or other physician.

### Follow up, outcome and covariates

Resident population was followed up for 4 years. Vital status and migration information were obtained from civil registry. Follow up began on January 1^st^ 2010 and continued until cancer diagnosis, death, emigration or end of follow up (i.e. December 31^st^ 2013), whichever came first.

Outcome of interest was cancer incidence rate, using the first incident cancer of each site during follow up and identifying it according to Reggio Emilia cancer registry. Only the first cancer was taken into account to calculate the all-sites cancer incidence; therefore, the sum of number of cases for each investigated cancer site turned out higher. The Reggio Emilia cancer registry includes all malignant cancer cases diagnosed in the Reggio Emilia province between January 1st 1996 and December 31^st^ 2013. Cancer site was coded according to International Statistical Classification of Diseases and Related Health Problems, 10th Revision. Non-melanoma skin cancers (C44), chronic myeloproliferative disorders, and myelodysplastic syndromes (D45-D47) were ruled out, according to the International rules of cancer registries.

Our analysis considered sex, age and foreign status as covariates.

### Statistical analysis

Baseline characteristics of study population were introduced as absolute numbers and percentages or as medians and interquartile range (IQR) and they were stratified by sex and diabetes status.

We calculated Incidence Rate Ratios (IRRs) and 95% Confidence Intervals (95% CI) using the multivariate Poisson regression model for all-sites and for specific investigated sites. Subjects without diabetes were considered as reference group. We did not define a threshold of significance, thus implying that no calculation of statistical power was made. Moreover we did not propose any statistical test. Confidence interval and *p*-values were only introduced to provide a measure of the likelihood that the differences were due to chance. *P*-values for IRR heterogeneity between sex and diabetes were calculated using log-likelihood ratio distribution. As latent cancer might have contributed to develop diabetes, we duplicated some analyses just for subjects with at least 2 years of diabetes duration at baseline, in order to reduce reverse causality. Furthermore, we estimated the IRR and 95% CI according to type of diabetes, diabetes duration at baseline (0–1 years; 2–5; 6–10; > 10), and treatment (the latter only for Type 2 diabetes). All models were adjusted by age at baseline, foreign status and sex (when not stratified). Finally, we compared risk among different types of treatment for Type 2 diabetes, using untreated patients (i.e. with diet-only and physical activity-only programs) as reference, and adding years since diagnosis at baseline (as continuous variable) to other covariates. Analyses were performed by use of STATA statistical package Version 12.0.

## Results

Study cohort included 407,157 subjects (Table 1): out of them, 23,358 had diabetes (5.7% resident population), 13,089 males and 10,269 females (6.5% male crude prevalence and 5.0% female crude prevalence). Median age and percentage of subjects with previous cancer were higher among diabetic population compared to non-diabetic one. The proportion of foreigners was twice as high in non-diabetic population. Subjects with Type 2 diabetes accounted for 96% total population with diabetes, and more than 75% of them were not on insulin therapy.

**Table 1:** Characteristics of the study cohort by sex and diabetes status

Characteristics	Total		Females		Males	
	Without DM	With DM	Without DM	With DM	Without DM	With DM
At baseline:						
Population 20–84 years	383,799	23,358	195,930	10,269	187,869	13,089
Foreigners^a^: N (%)	53,852 (14.0)	1812 (7.7)	28,103 (14.3)	862 (8.3)	25,749 (13.7)	950 (7.3)
Age (years): median (IQR)	45 (35–60)	67 (58–75)	47 (35–62)	69 (60–76)	44 (34–59)	66 (57–74)
History of cancer N (%)	12,685 (3.3)	1979 (8.5)	6878 (3.5)	840 (8.2)	5807 (3.1)	1139 (8.7)
Time since diabetes diagnosis (years) median (IQR)	*4 (1–10)*			*4 (1–10)*		*4 (1–10)*
Type of diabetes N(%)						
* Type 1 diabetes*		*779 (3.3)*		*364 (3.5)*		*415 (3.2)*
* Type 2 diabetes*		*22,458 (96.2)*		*9853 (96.0)*		*12,605 (96.3)*
* Secondary diabetes* ^*b*^		*121 (0.5)*		*52 (0.5)*		*69 (0.5)*
Treatment N(%)^c^						
* Diet only* ^d^		*6139 (27.3)*		*2636 (26.7)*		*3503 (27.8)*
* OHA* ^*e*^		*11,980 (53.3)*		*5243 (53.2)*		*6737 (53.4)*
* Insulin only*		*2313 (10.3)*		*1010 (10.2)*		*1303 (10.3)*
* OHAs + insulin*		*2026 (9.0)*		*964 (9.9)*		*1062 (8.4)*
During follow-up:						
Person-years	1,499,890	85,953	767,174	38,171	732,716	47,782
Dead: N (%)	9875 (2.6)	2704 (11.6)	4291 (2.2)	1071 (10.4)	5584 (3.0)	1633 (12.5)
Moved: N (%)	345 (0.1)	12 (0.1)	148 (0.1)	8 (0.1)	197 (0.1)	4 (0.0)

^a^taking into account the country of birth; ^b^diseases of the exocrine pancreas and drug-induced diabetes ^c^only for Type 2 diabetes; ^d^patients controlled only through diet and physical activity; ^*e*^oral hypoglycaemic agents. *IQR* Inter-quartile range

During follow-up, the percentage of deaths in diabetic population were four-fold in comparison to those in non-diabetic population, while the percentage of people relocated out of Reggio Emilia province was similar and very low, in both groups.

We identified 9858 first malignant cancer cases in non-diabetic population and 1464 cases in patients with diabetes (Table 2).

**Table 2:** No. of subjects with cancer by diabetes status, Incidence Rate Ratios (IRR) and 95% Confidence Intervals (95%CI) for subjects with diabetes vs subjects without diabetes

Cancer site^a^	Total				Females				Males			
	No DM	DM	IRR	95% CI	No DM	DM	IRR	95% CI	No DM	DM	IRR	95% CI
All sites	9858	1464	1.22	1.15–1.29	4851	563	1.25	1.15–1.37	5007	901	1.17	1.05–1.39
C16: Stomach	396	58	0.95	0.72–1.26	139	20	1.17	0.72–1.88	257	38	0.87	0.66–2.21
C18-C20: Colon-rectum	956	177	1.32	1.12–1.55	455	59	1.12	0.85–1.49	501	118	1.44	1.25–2.60
C22: Liver	207	99	3.37	2.63–4.32	61	23	3.26	2.00–5.35	146	76	3.40	2.40–7.16
C24: Biliary tract	69	16	1.41	0.81–2.45	28	7	1.76	0.76–4.04	41	9	1.22	0.07–4.58
C25: Pancreas	340	101	2.00	1.60–2.51	155	48	2.49	1.79–3.46	185	53	1.68	0.65–2.61
C33-C34: Lung	1047	177	1.10	0.93–1.29	329	36	1.01	0.71–1.43	718	141	1.11	0.67–1.47
C50: Breast	1643	148	1.05	0.89–1.26	1633	147	1.06	0.89–1.26	10	1	-	-
C54: Corpus uteri	-	-	-	-	249	44	1.84	1.33–2.56	-	-	-	-
C56: Ovary	-	-	-	-	139	21	1.56	0.97–2.49	-	-	-	-
C61: Prostate	-	-	-	-	-	-	-	-	938	134	0.86	0.72–1.04
C64-C66; C68: Kidney	377	60	1.20	0.67–2.14	111	19	1.55	0.94–2.54	266	41	1.02	0.74–1.44
C67;D09: Bladder	627	138	1.39	1.16–1.68	126	25	1.72	1.11–2.66	501	113	1.33	1.08–1.64
C73: Thyroid	504	27	1.00	0.67–1.49	368	18	1.05	0.64–1.70	136	9	0.88	0.44–1.77
C82-C85; C96: NHL^b^	425	56	1.09	0.82–1.44	180	20	1.14	0.71–1.82	245	36	1.05	0.74–1.50
Other sites^c^	2252	277	1.09	0.96–1.23	1001	102	1.11	0.89–1.36	1251	175	1.07	0.91–1.25

^a^Only first primary cancers are listed. Non-melanoma skin cancer (C44), chronic myeloproliferative disorders and myelodysplastic syndromes (D45-D47) were not counted as a cancer diagnosis; ^b^Non-Hodgkin lymphoma; ^c^Cancers not in any mentioned group. IRR = calculated using Poisson model, adjusted for age, foreign status, and sex (when no stratified). People without diabetes were used as reference

Prevalent diabetes at baseline was positively associated with total cancer incidence; the risk was slightly higher in females than in males (test for heterogeneity *P* = 0.0048). An increased risk for diabetic population was observed for liver, bladder, pancreas, and colorectal cancers. The pancreatic cancer excess risk seemed to be more evident in females than in males (heterogeneity *P* = 0.1041), while colorectal cancer was almost exclusively detected in males (test for heterogeneity *P* = 0.0672). Excess risk was found in corpus uteri. We found also an excess risk for ovary and kidney cancer in females that we cannot exclude to be random. On the other hand, as concerned prostate cancer, we observed a slightly lower risk in diabetic subjects than in non-diabetic ones, although this may also be random. After ruling out prostate cancer from the total male cancer category, excess risk slightly increased (IRR = 1.24, 95%CI 1.15–1.34), with values very close to female risk.

Focusing exclusively on subjects with at least 2 years of diabetes duration at baseline (*N* = 16,715), we observed risk ratios which were close to those found in the previous analysis (IRR = 1.21, 95%CI 1.11–1.31 for males and IRR = 1.28, 95%CI 1.16–1.42 for females). However, this sub-analysis did confirm excess female risk for kidney cancer, but not for ovary cancer. Additional file 1shows the full list of risk ratios by cancer for patients with diabetes with at least 2 years of diabetes duration at baseline.

Type 1 diabetes subjects showed a risk of developing a malignant neoplasm which was similar to that in non-diabetics population, although the few observed cases produced a wide confidence interval that included the 1.22 risk ratios observed for Type 2 diabetes (Table 3). With regard to Type 2 diabetes, we observed a slightly increased risk for untreated patients (i.e. just controlled through diet and physical activity), an intermediate excess risk for patients treated with OHAs only, and a more marked excess risk in insulin-treated patients, especially those treated with combined insulin-OHAs drugs.

**Table 3:** Population, No. of cancer, Incidence Rate Ratios (IRR) and 95% Confidence Intervals (95% CI) for type of diabetes, treatment (only for type 2 diabetes), and diabetes duration vs subjects without diabetes

	Person-years	N cancer	IRR	95% CI
Without diabetes	1,499,890	9858	1.00	-
With diabetes	85,953	1464	1.22	1.15–1.29
By type of diabetes:				
Type 1 diabetes	3017	15	0.88	0.53–1.47
Secondary diabetes^a^	393	10	2.04	1.10–3.80
Type 2 diabetes	82,542	1439	1.22	1.15–1.29
*By treatment:*				
* Diet only*	*22,900*	*349*	*1.10*	*1.00–1.23*
* OHAs only*	*44,637*	*792*	*1.22*	*1.14–1.32*
* Insulin only*	*7738*	*161*	*1.32*	*1.13–1.54*
* OHAs + insulin*	*7267*	*137*	*1.37*	*1.16–1.62*
By diabetes duration (years):				
0–1	24,553	361	1.10	0.99–1.23
2–5	24,710	403	1.23	1.11–1.36
6–10	17,033	337	1.44	1.29–1.61
11+	19,658	363	1.15	1.04–1.30

IRR = calculated using Poisson model, adjusted for age, foreign status, and sex. People without diabetes were used as reference. ^a^diseases of the exocrine pancreas and drug-induced diabetes

Considering only Type 2 diabetes population, and adjusting for length of time since diabetes diagnosis, we obtained similar results. Using untreated patients as reference, OHAs treated patients showed an IRR = 1.13 (95%CI 0.99–1.28), insulin treated patients an IRR = 1.27 (95%CI 1.04–1.55) and patients in therapy an IRR = 1.28 (95%CI 1.04–1.57).

Diabetes duration analysis showed an increasing risk until 6–10 years and a subsequent decrease to moderate-higher risk.

## Discussion

Our population-based cohort study showed an excess of cancer incidence risk in people with diabetes. The effect was appreciable only in Type 2 diabetes, while Type 1 diabetes cancer incidence was similar to that of the population without diabetes. Among subjects affected by Type 2 diabetes, association was more relevant for insulin-treated patients, especially for combined therapy users. Compared to previous studies, our study observed smaller overall excess, probably due to the population-based approach related to diabetes registry. It allocated all diabetic subjects to the exposed group, but not limited to patients treated in specialized care centres, as they may represent a selected population suffering from more severe diabetes. Moreover, risk ratios did not substantially decrease when our analysis included only subjects with at least 2 years of diabetes duration. The algorithm used in Reggio Emilia our diabetes registry could detect diabetes subjects since disease onset, thus reducing potential detection bias, i.e. increasing likelihood of cancer diagnosis during diabetes initial assessment and follow up, as some authors assumed [17].

Nevertheless, we noted that relevant excess for some cancer sites was highly unlikely to be related to random fluctuations. In particular, our study turned out to be consistent with previous studies suggesting an increased cancer incidence for liver [4, 18], pancreas [5], colon rectum [11], and bladder [10] in population with diabetes. Furthermore, we found excess cancer risk for corpus uteri [6, 18], and a suggestion of reduced prostate cancer incidence [9, 18]. Finally, our data suggested an increased risk for ovary and kidney cancer in females, although increased risk for ovary cancer disappeared narrowing the analysis to subjects with at least 2 year diabetes duration.

A recent meta-analysis on diabetes and kidney cancer incidence [7] has suggested a stronger association in women, although there have been claims that “different proportions of men and women in the studies may in part account for the observed heterogeneity” and that obesity, which is more prevalent in women than men, could be a potential confounder.

As concerns pancreas cancer, the literature showed inconsistent results, and some studies have reported an up 4–5 fold increased risk for diabetic patients, while other studies did not find any increase at all [5]. It must be stressed that studies which could effectively rule out reverse causality, (i.e. cancer increasing the risk of diabetes rather than vice versa) found a moderate increase of pancreas cancer risk in people with diabetes. Our study found an excess risk for pancreas, also restricting the analysis to patients with at least 2 years of diabetes duration, in which case reverse causality overcame.

### Association with hypoglycaemic agents

As concerns Type 2 diabetes, we had an opportunity of classifying diabetic population according to antidiabetic treatment during 2009. We observed an increased risk related to increased therapy complexity, an indicator of disease severity. Our results were confirmed by the analysis performed among Type 2 diabetes subjects where we use also time since diagnosis as covariate. Unfortunately, we could not define treatment duration and consequently disentangle the insulin effect on cancer initiation and possible masked worse metabolic conditions (indication bias) or possible close monitoring practice (detection bias), as some authors did [17]. Insulin association was consistent with other studies, which suggested a direct role of exogenous insulin and insulin analogues in carcinogenicity [19–22]. On the other hand, a direct role of insulin was inconsistent with the absence of any increase in cancer risk in Type 1 diabetes patients, who experienced a much longer use of insulin in their life.

The highest IRR was detected in patients treated with both OHAs and insulin. Such therapeutic regimen is usually followed by patients who cannot reach their glycaemic target with the help of just OHAs, often as a preliminary step before initiating sole insulin therapy [23]. However, it can also be used in patients treated with insulin who gain weight or in patients with low compliance to insulin regimen. These patients may all show unstable and high glycaemic values or a worse metabolic state, so hyperglycaemia could amplify the hyperinsulinemia effect, thus increasing cancer risk.

Only a slight excess risk was observed in untreated Type 2 diabetes subjects, in comparison to drug-treated subjects. Such excess risk cannot be due to insulin or OHAs, rather, it might be confounded or mediated by overweight and obesity, which are well- known risk factors for both diabetes and many type of cancers [24].

### Diabetes duration

Our study detected an increasing risk for diabetes duration up to 10 years from diagnosis and a subsequent decrease to moderate-higher risk. Our cohort study, which included prevalent cases of diabetes and incident cases of cancer, possibly minimized the so-called indication bias, which was detected by other studies [17, 18] in which follow up started at diabetes onset and tests recommended after a diabetes diagnosis might have increased the probability of detecting prevalent asymptomatic cancers. A decreased relative risk in the last group of diabetic subjects could be due to higher cancer incidence in people with diabetes, leading to a decreased susceptible population. A similar phenomenon of decreased excess risk has been recorded observed for mortality [25] and for cancer [26].

### Strengths and limits

Strengths are represented by population-based approach thanks to clinically confirmed diabetes registry data, gender approach, including evaluation of heterogeneity between sexes and identification of the type of diabetes, including secondary diabetes, whose causality direction may be difficult to disentangle.

Classification according to patients’ current drug exposure could lead to misclassification. Moreover, we could not evaluate the potential role of some other non-assessed potential confounders, such as different types of insulin used (e.g. insulin analogues vs. human insulin), different mean daily doses of insulin, other drugs taken (e.g., acetylsalicylic acid, statins), glyco-metabolic control, and insulin resistance being present or not.

Nevertheless, the results related to “diet only” and “OHAs only” groups could be unaffected by insulin, and the former also by OHA response. Moreover, our cross-sectional classification of drug exposure was likely to be unaffected by immortal time bias (as we do not set conditions on the exposure duration) and by time-window bias (as we made no use of time dependent variables for exposure).

We did not collect relevant data about other risk factors, in particular positive family history for cancer, smoking, alcohol consumption, physical inactivity, BMI, workplace exposure to toxic substances, etc. We carried out a population-based study and, unlike observational studies of patients using clinical databases, we had limited clinical information on the general population. As regards smoking attitude, our data were confirmed by absence of lung cancer excess. On the other hand, we could not adjust for BMI, although we are well aware that BMI and diabetes could share some mechanisms of cancerogenesis or they could be two rings in the same causal chain. Therefore, further studies should be conducted to analyse possible mediation effect of glucose metabolism in the relationship between BMI and cancer.

As we mainly examined Caucasian race, it was impossible to extend our results to other ethnicities, although our analyses took into account foreign status.

Finally, we considered just 4-year follow up. Such time duration may seem quite short, if compared to time lag from exposure and cancer onset for most cancer sites. Thus, we were mainly concentrated on the effect of diabetes exposure occurred in the years before we started follow up.

## Conclusions

This observational study, which was carried out in an Italian province using population-based registries, found an overall 15–30% higher cancer incidence among subjects with diabetes in comparison to those without diabetes. Excess cancer risk persisted, when we restricted our analysis to patients with at least 2-years of diabetes duration. Cancer sites driving the overall increased risk were: liver, pancreas, bladder, and colon-rectum, corpus uteri for females, regardless of diabetes duration. There was also suggestion for an increased risk for kidney cancer in women and a decreased risk for prostate cancer. Compared to non-diabetic population, the excess risk was ppreciable for Type 2 diabetes. Insulin, monotherapy or combined therapy (per se or as indication of poor blood glucose control) and diabetes duration may play a role in the association between diabetes and cancer.

Considering high and increasing prevalence of diabetes, a slightly increase incidence among population with diabetes could have a strong impact on burden of cancer at population level.

## Additional file

Additional file 1: No. of subjects with cancer by diabetes status, Incidence Rate Ratios (IRR) and 95% Confidence Interval (95%CI) for subjects with at least 2 years of diabetes duration vs subjects without diabetes. Cancer incidence risk analysis for subjects with at least 2 years of diabetes duration compared to subjects without diabetes. (DOC 52 kb)

## Abbreviations

OHA: Oral hypoglycaemic agents
