# Supplementary material for: Diabetes and risk of cancer incidence: results from a population-based cohort study in northern Italy
Source: BMC Cancer. 2017 Oct 25;17:703. doi: 10.1186/s12885-017-3696-4 (PMC5657107; doi:10.1186/s12885-017-3696-4)
Supplement: Additional file 1: — No. of subjects with cancer by diabetes status, Incidence Rate Ratios (IRR) and 95% Confidence Interval (95%CI) for subjects with at least 2 years of diabetes duration vs subjects without diabetes. Cancer incidence risk analysis for subjects with at least 2 years of diabetes duration compared to subjects without diabetes. (DOC 52 kb) [file 12885_2017_3696_MOESM1_ESM.doc]

**Annex 1 No. of subjects with cancer by diabetes status, Incidence Rate Ratios (IRR) and 95% Confidence Intervals (95%CI) for subjects subjects with at least two years of diabetes duration vs subjects without diabetes**

|  | **Total** | | | |  | **Females** | | | |  | **Males** | | | |
| --- | --- | --- | --- | --- | --- | --- | --- | --- | --- | --- | --- | --- | --- | --- |
| **Cancer site^a^** | **No DM** | **DM** | **IRR** | **95% CI** |  | **No DM** | **DM** | **IRR** | **95% CI** |  | **No DM** | **DM** | **IRR** | **95% CI** |
| All sites | 9858 | 1103 | 1.26 | 1.18-1.34 |  | 4851 | 411 | 1.28 | 1.16-1.42 |  | 5007 | 692 | 1.21 | 1.11-1.31 |
| C16: Stomach | 396 | 47 | 1.05 | 0.78-1.43 |  | 139 | 17 | 1.40 | 0.85-2.31 |  | 257 | 30 | 0.92 | 0.63-1.35 |
| C18-C20: Colonrectum | 956 | 128 | 1.30 | 1.08-1.57 |  | 455 | 41 | 1.09 | 0.79-1.51 |  | 501 | 87 | 1.43 | 1.13-1.80 |
| C22: Liver | 207 | 82 | 3.77 | 2.89-4.90 |  | 61 | 18 | 3.61 | 2.11-6.18 |  | 146 | 64 | 3.81 | 2.81-5.16 |
| C24: Biliary tract | 69 | 16 | 1.92 | 1.11-3.33 |  | 28 | 7 | 2.46 | 1.07-5.66 |  | 41 | 9 | 1.64 | 0.79-3.49 |
| C25: Pancreas | 340 | 78 | 2.10 | 1.63-2.70 |  | 155 | 35 | 2.51 | 1.63-3.74 |  | 185 | 43 | 1.83 | 1.31-2.57 |
| C33-C34: Lung | 1047 | 139 | 1.16 | 0.97-1.39 |  | 329 | 25 | 0.98 | 0.65-1.47 |  | 718 | 114 | 1.20 | 0.99-1.47 |
| C50: Breast | 1643 | 113 | 1.13 | 0.93-1.37 |  | 1633 | 112 | 1.13 | 0.93-1.37 |  | 10 | 1 | - | - |
| C54: Corpus uteri | - | - | - | - |  | 249 | 32 | 1.87 | 1.28-2.72 |  | - | - | - | - |
| C56: Ovary | - | - | - | - |  | 139 | 15 | 0.62 | 0.91-2.70 |  | - | - | - | - |
| C61: Prostate | - | - | - | - |  | - | - | - | - |  | 938 | 99 | 0.86 | 0.69-1.05 |
| C64-C66; C68: Kidney | 377 | 53 | 1.38 | 1.03-1.85 |  | 111 | 15 | 1.71 | 0.99-2.95 |  | 266 | 38 | 1.28 | 0.91-1.81 |
| C67;D09: Bladder | 627 | 102 | 1.39 | 1.13-1.72 |  | 126 | 17 | 1.64 | 0.98-2.74 |  | 501 | 85 | 1.34 | 1.06-1.70 |
| C73: Thyroid | 504 | 21 | 1.09 | 0.70-1.70 |  | 368 | 15 | 1.23 | 0.72-2.08 |  | 136 | 6 | 0. 81 | 0.35-1.86 |
| C82-C85; C96: NHL^b^ | 425 | 39 | 1.04 | 0.74-1.44 |  | 180 | 12 | 0. 95 | 0.53-1.72 |  | 245 | 27 | 1.07 | 0.71-1.60 |
| Other sites^c^ | 2252 | 195 | 1.05 | 0.90-1.22 |  | 1001 | 72 | 1.10 | 0.86-1.40 |  | 1251 | 123 | 1.01 | 0.84-1.22 |

^a^Only first primary cancers are listed. Non-melanoma skin cancer (C44), chronic myeloproliferative disorders and myelodysplastic syndromes (D45-D47) were not counted as a cancer diagnosis; ^b^Non-Hodgkin lymphoma; ^c^Cancers not in any mentioned group. IRR = calculated using Poisson model, adjusted for age, foreign status, and sex (when not stratified). People without diabetes were used as reference.
